# Supplementary figures and images for: Protein Family Content Uncovers Lineage Relationships and Bacterial Pathway Maintenance Mechanisms in DPANN Archaea
Source: Front Microbiol. 2021 Jun 1;12:660052. doi: 10.3389/fmicb.2021.660052 (PMC8204110; doi:10.3389/fmicb.2021.660052)

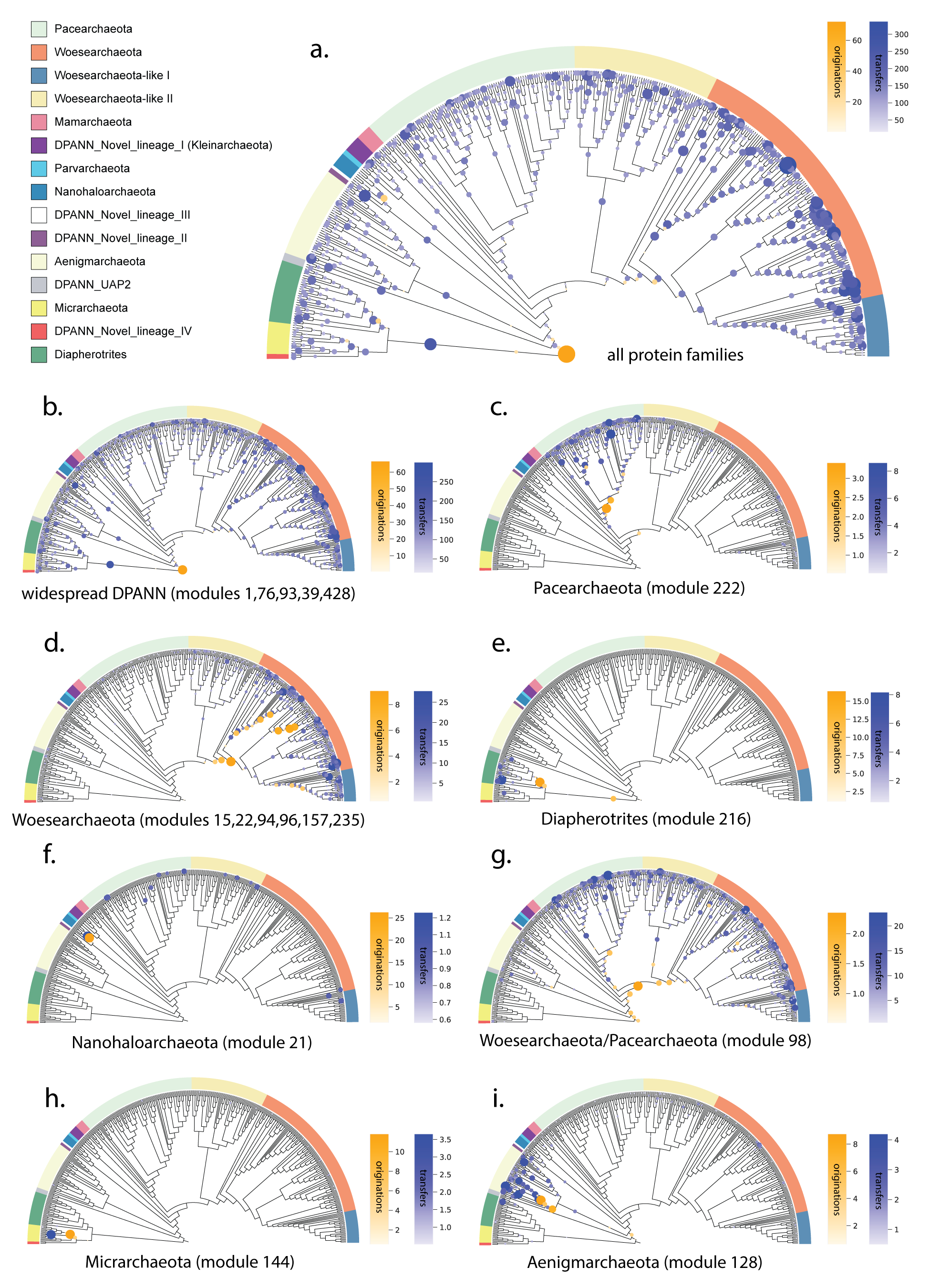

Supplement: Supplementary Figure 2 — Evolution of protein families in the DPANN archaea. Each panel displays the species tree of the 390 genomes used for the protein families analysis in cladogram format. The size and hue of circles represent the cumulative number of originations (orange) - defined as either lateral transfer from outside the lineages examined here, or de novo evolution - and intra-DPANN transfers (blue) predicted to occur on that branch. Evolutionary reconstructions are shown for gene families in all/widespread modules (panel ab) and lineage-specific modules detailed in Table 1 (panels c-i). [file Image_2.PNG]
