# Supplementary material for: Protein Family Content Uncovers Lineage Relationships and Bacterial Pathway Maintenance Mechanisms in DPANN Archaea
Source: Front Microbiol. 2021 Jun 1;12:660052. doi: 10.3389/fmicb.2021.660052 (PMC8204110; doi:10.3389/fmicb.2021.660052)
Supplement: Supplementary Figure 1 — Taxonomic assessment and distribution of the newly DPANN genomes with metabolic similarities and distribution among DPANN lineages. Maximum-likelihood phylogeny of the DPANN lineages using Altiarchaeota as an outgroup and based on a 14-ribosomal-protein concatenated alignment (3677 amino acids, LG+R10 model). The presence of targeted metabolic traits is indicated with colored boxes. The scale bar indicates the mean number of substitutions per site. Bootstrap values are also indicated at the branches. [file Image_1.PDF]

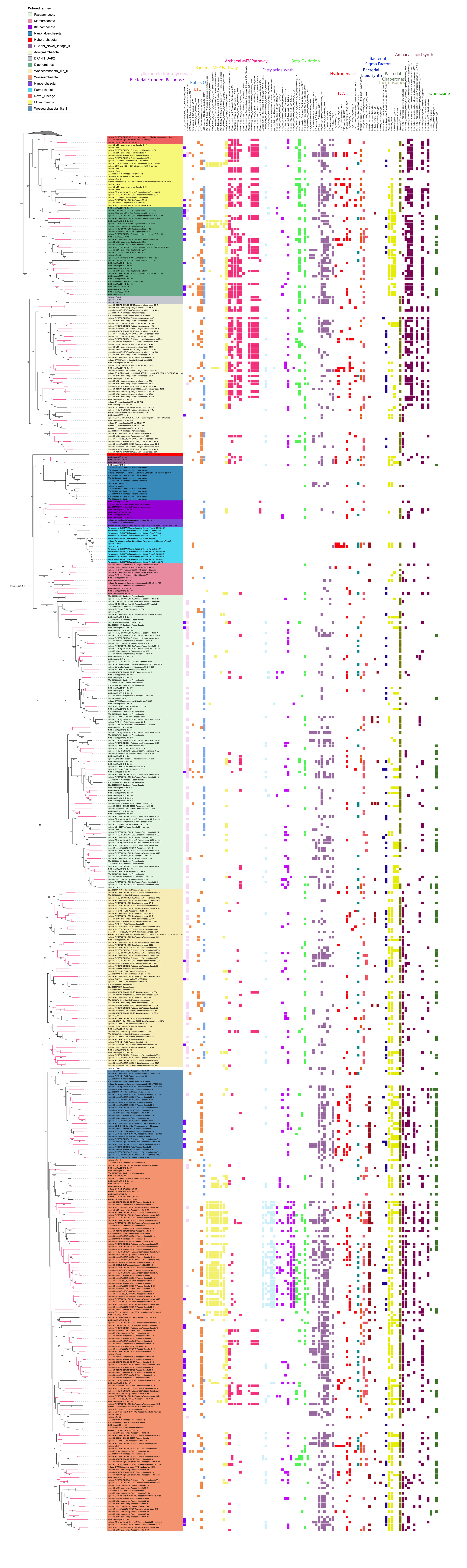

Colored ranges
Pacearchaeota
Mamarchaeota
Kleinarchaeota
Nanohaloarchaeota
Huberarchaeota
DPANN_Novel_lineage_II
Aenigmarchaeota
DPANN_UAP2
Diapherotrites
Woesearchaeota_like_II
Woesearchaeota
Nanoarchaeota
Parvarchaeota
Novel_Lineage
Micrarchaeota
Woesearchaeota_like_I
Archaeal MEV Pathway
Beta-Oxidation
Bacterial MEP Pathway
Fatty acids synth
Lytic murein transglycosylases
Bacterial Stringent Response
RubisCO
ETC
Hydrogenase
TCA
Bacterial Sigma Factors
Bacterial Lipid synth
Bacterial Chaperones
Thermosomes
Archaeal Lipid synth
Queuosine
